# Beyond CC398: Characterisation of Other Tetracycline and Methicillin-Resistant *Staphylococcus aureus* Genetic Lineages Circulating in Spanish Hospitals

**DOI:** 10.3390/pathogens11030307

**Published:** 2022-03-01

**Authors:** Sara Ceballos, Carmen Lozano, Carmen Aspiroz, Laura Ruiz-Ripa, Paula Eguizábal, Allelen Campaña-Burguet, Emilia Cercenado, Ana Isabel López-Calleja, Javier Castillo, Jose Manuel Azcona-Gutiérrez, Luis Torres, Jorge Calvo, Carmen Martin, María Navarro, Myriam Zarazaga, Carmen Torres, the Study Group of Clinical LA-MRSA

**Affiliations:** 1 Area Bioquímica y Biología Molecular, Universidad de La Rioja, 26006 Logroño, Spain; saraceballos@hotmail.es (S.C.); carmen.lozano@unirioja.es (C.L.); laura_ruiz_10@hotmail.com (L.R.-R.); paulaeguimar@gmail.com (P.E.); allelencampanaburguet@gmail.com (A.C.-B.); myriam.zarazaga@unirioja.es (M.Z.); 2 Servicio de Microbiología, Hospital Royo Villanova, 50015 Zaragoza, Spain; carmen.aspiroz@gmail.com; 3 Servicio de Microbiología y Enfermedades Infecciosas, Hospital General Universitario Gregorio Marañón, CIBERES (CIBER de Enfermedades Respiratorias), 28007 Madrid, Spain; emilia.cercenado@salud.madrid.org; 4 Hospital Universitario Miguel Servet/IIS Aragón, 50009 Zaragoza, Spain; ailopezcal@salud.aragon.es; 5 Hospital Clínico Universitario Lozano Blesa, 50009 Zaragoza, Spain; macarios@unizar.es; 6 Hospital San Pedro, 26006 Logroño, Spain; jmazcona@riojasalud.es; 7 Hospital San Jorge, 22004 Huesca, Spain; ltorres@salud.aragon.es; 8 Hospital Universitario Marqués de Valdecilla, CIBERINFEC (CIBER de Enfermedades Infecciosas, Instituto de Salud Carlos III), 39008 Santander, Spain; jorge.calvo@scsalud.es; 9 Complejo Hospitalario de Navarra, 31008 Pamplona, Spain; carmen.martin.salas@navarra.es; 10 Hospital Universitari de Vic, 08500 Vic, Spain; mnavarro@chv.cat

**Keywords:** MRSA, tetracycline resistance, CC398, CC1, t127, multicentre study

## Abstract

Tetracycline resistance (Tet^R^) has been evidenced as a good phenotypic marker for detection of livestock-associated methicillin-resistant *Staphylococcus aureus* (LA-MRSA) isolates of the clonal complex CC398. The aim of this study was to characterise a collection of 95 Tet^R^-MRSA isolates, not belonging to the lineage CC398, that were obtained in a previous multicentre study, to detect other MRSA clonal complexes that could be associated with this phenotypic Tet^R^ marker. The Tet^R^-MRSA isolates were recovered from 20 Spanish hospitals during 2016 and they were characterised to determine their antimicrobial resistance and virulence phenotypes/genotypes as well as the presence of the immune evasion cluster (IEC). A high proportion of isolates belonging to the CC1 lineage (46%) were observed, as well as to the CC5, CC8 and CC45 lineages (11% each one). Thirty-two different *spa*-types were identified, being predominantly CC1-t127 (40%) and CC45-t1081 (11%). The IEC system (with the gene *scn* as marker) was present in 73% of isolates and 16% produced the Panton Valentine leucocidin (PVL). A high proportion of MRSA-CC1 isolates were *scn*-negative (38.6%) and 52.9% of them were *blaZ*-negative. A multidrug resistance (MDR) phenotype was identified in 86% of MRSA isolates. The knowledge of other Tet^R^-MRSA genetic lineages, in addition to CC398, is highly relevant, since most of them were MDR and some of them presented important virulence factors. Strains potentially associated with livestock (as the subpopulation CC1-t127-*scn*-negative) or with humans (as the CC45 lineage or the subpopulation CC1-*scn*-positive) have been found in this study. The use of tetracycline-resistance for detection, not only of CC398 but also of other LA-MRSA lineages should be tracked in the future.

## 1. Introduction

*Staphylococcus aureus* is a common bacterium in human and animal regular microbiota, particularly present in the nose and on the skin. However, *S. aureus* can be an important opportunistic pathogen capable of causing anything from mild skin to life-threatening infections. *S. aureus* is also able to acquire multiple antimicrobial resistance mechanisms, and standing out amongst them, methicillin-resistant *S. aureus* (MRSA) isolates because of its resistance to almost all β-lactams [1].

Different clonal lineages have been initially proposed to be directly related to the environment in which they are found. In this way, we classically found hospital-associated (HA) or community-acquired (CA) MRSA. Nevertheless, nowadays this line of distinction within groups is becoming more and more blurred.

Newer livestock-associated (LA) MRSA lineages, genetically different from human isolates and closely related to farm animals, have been found more regularly in the healthcare system [2]. During the last decade, livestock have been considered to be a great reservoir of MRSA with high potential of zoonotic transmission to humans [3]. It has been observed that the spread of LA-MRSA from animals to humans occurs frequently and that livestock workers present a high risk for LA-MRSA colonisation and subsequent infection [4]. The abusive use of antimicrobials like tetracycline in food production animals leads to the selective pressure of tetracycline-resistant (Tet^R^) LA-MRSA isolates [5]. The most common LA-MRSA lineage in Europe belongs to the clonal complex CC398. Tet^R^ is one of its most outstanding features, a few atypical tetracycline-susceptible LA-MRSA strains have been detected [6], and this phenotype is commonly used for its detection [7]. LA-MRSA CC398 is related to pig farming and contact with these animals seems to be the driving force for human transmission. However, more and more often the frequent appearance of LA-MRSA cases in people without livestock contact is being reported [7].

Other LA-MRSA genetic lineages have been also related to animals and they are gaining relevance in the public health landscape [8,9,10,11]. While some of them have been also associated with pig farming (CC1, CC9 or CC97), others have been found in cattle (CC130, CC425), horses (CC8) and/or poultry (CC5) [12]. Nevertheless, frequently many of these clonal lineages have been detected in different animal hosts [12] and transmission among different livestock animals has been suggested [13].

In a previous study, a collection of 232 Tet^R^-MRSA isolates was obtained during a six-month period from 20 Spanish hospitals located in regions with different pig farming densities, in order to seek for potential CC398 isolates. Almost 60% of the Tet^R^-MRSA isolates corresponded to the lineage CC398 (n = 137 isolates), and a significant correlation was evidenced between the rate of MRSA CC398 detected in a hospital and the pig farming density in the adjoining region [14]. The non-CC398 isolates of this collection (n = 95) were not further characterised, and the unique data known were that 46% of them corresponded to the clonal complex CC1 [14]. The Sequence Type (ST) 1 belonging to CC1 lineage, producer of the Panton–Valentine leucocidin (PVL), is well recognised as a CA-MRSA in human infections, also known as USA400, and common in the USA [15]. In Europe, some cases of this PVL-positive clone have been reported, for instance in Italy [16] or Denmark [17]. Nevertheless, a different CA-MRSA ST1 subpopulation (presenting the *spa*-type t127) has been described in the United Kingdom, highlighting its lack of PVL production [18,19]. The *spa*-type t127 has been associated with LA-MRSA isolates and, when recovered from pigs, the isolates are generally IEC negative [20]. However, some studies have reported the presence of MRSA *spa*-type t127 in isolates of farmed animal origin (especially of bovine) with similar characteristics to the human clade, such as the presence of the genes *scn*, *sak*, *sea* or *sat* [20,21]. Several reports have highlighted the presence of MRSA-CC1-t127 isolates colonising pigs and also colonising or causing infections in people in contact with these farm animals [9,11,22].

Considering the results obtained in the previous study, in which Tet^R^ was evidenced as a good phenotypic marker for LA-MRSA detection of the lineage CC398, the possibility of finding other LA-MRSA clonal lineages was analysed. The aim of the present study was to carry out the complete characterisation of the referred collection of the 95 non-CC398 Tet^R^-MRSA isolates by analysing their antimicrobial resistance and virulence phenotypes and genotypes, as well as the presence of the IEC system and their corresponding clonal lineages.

## 2. Results

### 2.1. Molecular Characterisation of MRSA Isolates

Thirty-two different *spa*-types were identified among the 95 Tet^R^-MRSA isolates analysed (Table 1 and Table 2), with a40% of them belonging to *spa*-type t127, followed by t1081 (10.5%), t148 (6.3%), t002 (5.3%), and others with only one to three isolates per *spa* type (37.9%). Three new *spa*-types were detected in this study: t17234, t1723, and t17236.

The *spa* types were ascribed to nine different clonal complexes (CC1, CC5, CC7, CC8, CC30, CC45, CC80, CC88, CC121), and to two sequence types (ST2625 and a ST registered as ST5427, a single-locus variant of ST2050 with a different *aroE* allele) (Table 1 and Table 2). The most prevalent CC was CC1 (46%), including the following *spa*-types (number of isolates): t127 (38), t1381 (2), t174 (1), t693 (1), t1784 (1), and t2207 (1) (Table 3). The CC1 was present in 14 out of the 17 participant hospitals (Table 1). The SCC*mec* type was analysed in all 38 CC1-t127 isolates and all of them belonged to type IVa.

### 2.2. Sample Origin

According to the origin of MRSA isolates, 80% were obtained from clinical samples and the remaining 20% from epidemiological surveillance (ES) samples. Within clinical isolates, 64.5% were obtained from samples of skin and soft tissue infections (SSTI), 18.4% from respiratory tract infections (RTI), 5.3% from surgical site infections (SSI), 6.5% from urinary tract infections (UTI) and 5.3% from blood. Table 2 shows the distribution of the different sample origins according to the *spa*-types detected in the study.

### 2.3. Presence of the IEC and PVL Toxin

**Figure 1:**
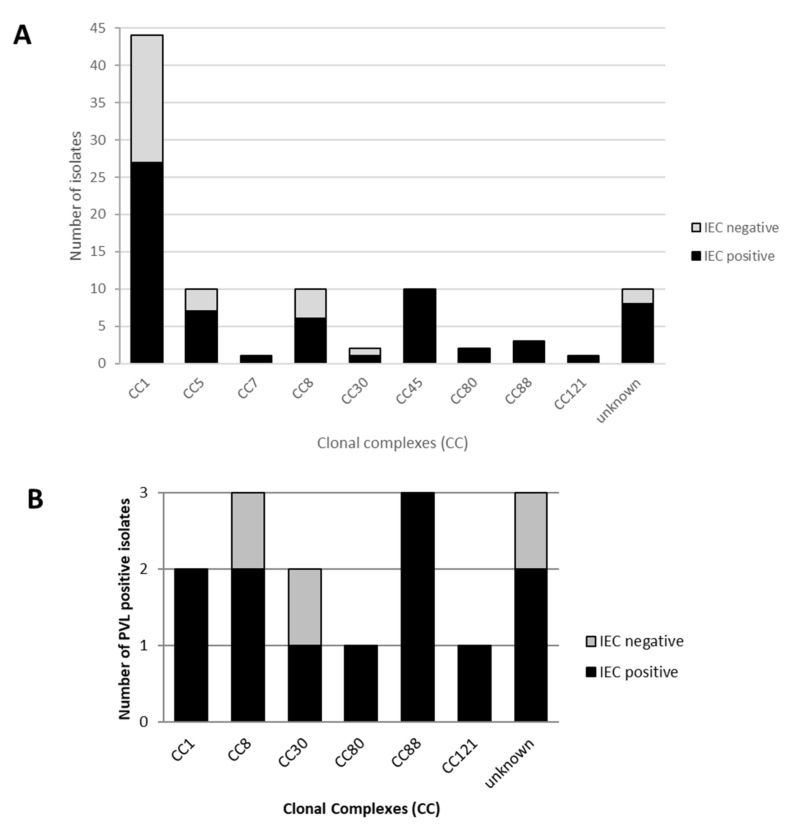
Presence of immune evasion cluster (IEC) among Tet^R^-MRSA non-CC398 isolates: (**A**) Presence or absence of IEC according to the clonal complexes (CCs) of the isolates; (**B**) Presence or absence of the IEC in PVL-positive isolates according to their clonal complexes (CCs).

**Figure 2:**
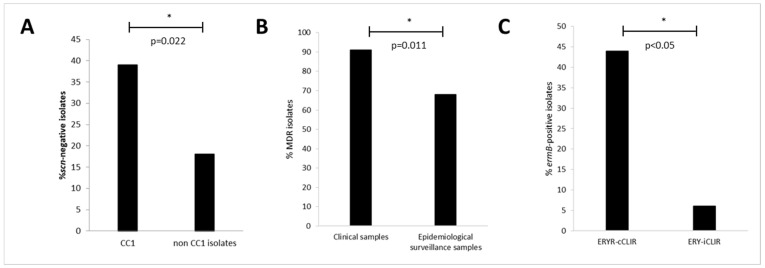
Statistically significant differences found in the characterisation of our Tet^R^-MRSA non-CC398 isolates. (**A**) Absence of *scn* in CC1 and non-CC1 isolates; (**B**) MDR detection in clinical and epidemiological surveillance samples; (**C**) Detection of the *ermB* gene in erythromycin-clindamycin constitutive resistant (ERY^R^-cCLI^R^) and erythromycin-clindamycin (ERY^R^-iCLI^R^) inducible resistant isolates. * *p* ≤ 0.05.

The IEC system (*scn*-positive) was present in 69/95 (73%) MRSA isolates analysed (Table 1 and Table 2). The CC1, CC5, CC8 and CC30 lineages contained both IEC-positive and IEC-negative isolates. However, all CC45, CC80, CC88 and CC121 lineages included only IEC-positive isolates (Figure 1A). Considering only the 38 isolates with the prevalent CC1- t127 lineage, 39% were *scn*-negative and, of them, 60% were recovered from SSTI (compared to *scn*-positive isolates where 34% were IPPB) (Table 3). There was a statistically significant difference (*p* = 0.022) between the proportion of *scn*-negative isolates considering CC1 isolates and the rest of isolates included in other CCs (Figure 2A).

The PVL virulence factor was detected in 15 MRSA isolates (16%) (Table 2 and Figure 1B), with 40% of PVL-producer isolates recovered from SSTI, 27% from RTI and 13% from ES samples, among others (Table 2). Only two CC1 isolates (*spa*-types t127 and t1784) were PVL-positive (Table 3). Moreover, three PVL-positive isolates were negative for the IEC, these isolates belonging to CC8-t148, CC30-t665 and t4725 (Figure 1B).

### 2.4. Multidrug Resistance (MDR) Phenotypes

**Figure 3:**
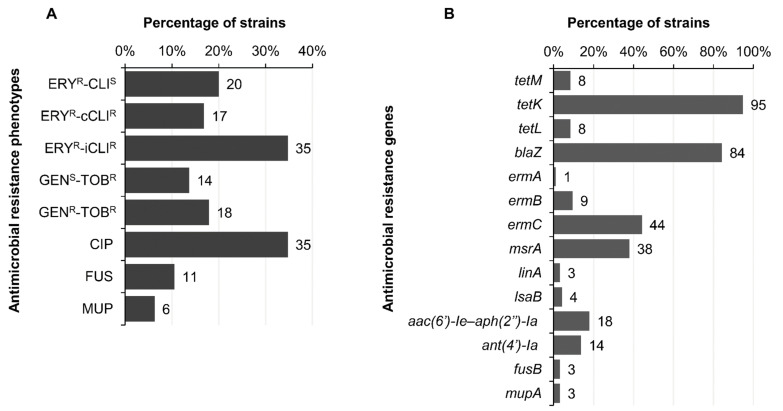
Antimicrobial resistance phenotype and genotype detected among Tet^R^-MRSA non-CC398 isolates. (**A**) Antimicrobial resistance phenotype (ERY, erythromycin; CLI, clindamycin; CIP, ciprofloxacin; FUS, fusidic acid; MUP, mupirocin); (**B**) Antimicrobial resistance genotype.

The collection of MRSA isolates analysed was resistant to different groups of antimicrobial agents (Figure 3A). A MDR phenotype was present in 86% (82/95) of studied MRSA isolates, with 24% of them resistant to three different families, 38% resistant to four, 18% resistant to five, 16% resistant to six and 4% resistant to seven or more different antimicrobial families. Among non-multidrug resistant isolates, 13 isolates were resistant to β-lactams and tetracycline and 69% of them belonged to lineage CC1 (Table 3).

There were statistically significant differences (*p* = 0.011) between clinical and ES MRSA isolates in terms of the MDR phenotype rates (Figure 2B). On the other hand, the differences when comparing the MDR phenotypes between the CC1 isolates and the remaining isolates were not statistically significant (*p* > 0.05).

None of the 95 analysed MRSA isolates was resistant to trimethoprim/sulfamethoxazole, vancomycin, teicoplanin, linezolid, daptomycin or chloramphenicol.

### 2.5. Antimicrobial Resistance Phenotypes and Genotypes

**Figure 4:**
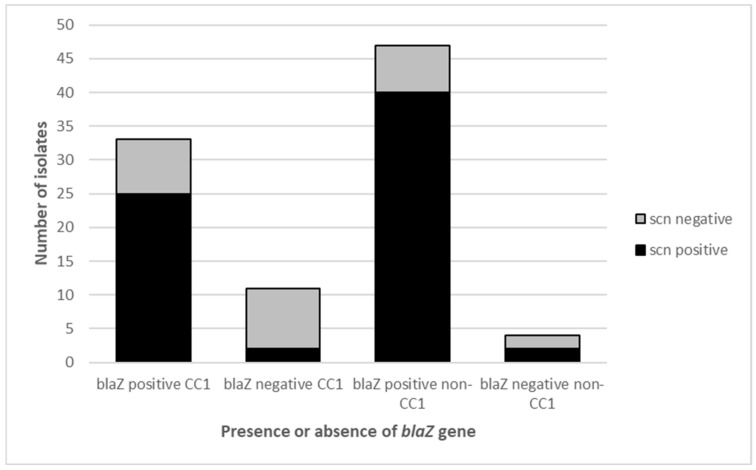
Presence or absence of the resistance *blaZ* gene in CC1 and non-CC1 tetracycline-resistant MRSA isolates according to the presence or absence of the IEC (*scn* gene).

As known previously, all our MRSA isolates were *mecA* carriers, conferring methicillin resistance [14]. In addition, 84% of isolates (n = 80) harboured the *blaZ* gene, encoding a penicillinase (Figure 3B); the absence of the *blaZ* gene was more frequent among MRSA *scn*-negative isolates (11/26, 42.3%) than among *scn*-positive isolates (4/69, 5.8%). Moreover, 52.9% (9/17) of MRSA-CC1-*scn*-negative isolates were *blaZ*-negative, and only 7.4% (2/27) of CC1-*scn*-positive isolates were *blaZ*-negative (Figure 4).

All of the MRSA isolates were Tet^R^, as it was a criterion for the isolates’ selection, and 95% of them carried the *tetK* gene (Figure 3B), usually alone (82/95) or in combination with *tetL* (4/95), *tetM* (3/95) or both genes (1/95). The remaining 5% Tet^R^-MRSA isolates presented the *tetM* gene alone (one isolate t2849 and one t1954) or *tetL* alone (one t008 isolate); or both genes (two t17235 isolates).

Regarding macrolides and lincosamides, 72% of MRSA isolates showed resistance (Figure 3A), with three different phenotypes detected: (a) 19 MRSA isolates (20%) showed resistance to erythromycin but not to clindamycin (ERY^R^-CLI^S^); (b) 16 isolates (17%) exhibited the constitutive erythromycin-clindamycin resistance (ERY^R^-cCLI^R^) phenotype; and (c) 33 isolates (35%) presented the inducible erythromycin-clindamycin resistance (ERY^R^-iCLI^R^) phenotype. The phenotype ERY^S^-CLI^R^, very frequent among MRSA-CC398 isolates [14], was not detected among the isolates of this study. The resistance genes related to macrolide-lincosamide resistance detected in this study are shown in Table 4.

Isolates with ERY^R^-iCLI^R^ phenotype carried more frequently the *ermC* gene (91%) compared with ERY^R^-cCLI^R^ isolates (75%), but no statistically significant differences between both groups were found (*p* > 0.05). On the other hand, ERY^R^-cCLI^R^ isolates presented a higher rate of *ermB* gene (44%) in comparison with ERY^R^-iCLI^R^ group (6%), and, in this case, the statistical analysis showed a significant difference (*p* < 0.05) (Figure 2C). The *ermT* and *linB/vgaA* genes were not detected among our ERY^R^ and CLI^R^ isolates, respectively.

Considering the aminoglycosides, 32% of isolates showed resistance to this group. More specifically, 14% of analysed isolates showed resistance to tobramycin alone (GEN^S^-TOB^R^) and 18% of isolates did it in combination with gentamicin (GEN^R^-TOB^R^) (Figure 3A). The resistance gene found associated with the GEN^S^-TOB^R^ phenotype was in all cases *ant(4′)-Ia*, and for GEN^R^-TOB^R^ phenotype the *aac(6′)-Ie-aph(2″)-Ia* gene (Figure 3B).

The analysed non-CC398 MRSA isolates also presented resistance to ciprofloxacin in 35% of cases (33/95) (Figure 3A). In addition, three isolates presented the *fusB* gene for fusidic acid resistance, although ten isolates were resistant to this antimicrobial agent (11%) (Figure 3A,B). For mupirocin, 6% (6/95) of analysed isolates showed a resistance phenotype, and, of them, three isolates harboured the *mupA* gene (Figure 3A,B).

### 2.6. Analysis of a Possible Relationship between MRSA CC1 and Pig Farming Density of Adjoining Region

Spearman correlations between pig densities (estimated in pigs/km^2^ per each region in which hospitals are located) and different proportions of MRSA CC1 or MRSA t127 or *scn*-negative MRSA (respect to total number of MRSA, *S. aureus*, Tet^R^-MRSA or MRSA non-CC398) were analysed. For each pair of variables studied, a non-statistically significant association was detected (*p* > 0.05). Considering the population densities of each region (inhabitants/km^2^) and the proportions of MRSA CC1 analysed before, the results were similar, with no statistically significant association (*p* > 0.05).

## 3. Discussion

Tetracycline has been widely used for preventing and treating farm-animal infections, contributing with its use, and potential abuse, to the increase of antimicrobial resistance worldwide. Tet^R^ was evidenced as a good phenotypic marker for LA-MRSA detection in previous studies [7,14] In the present study, the Tet^R^-MRSA non-CC398 isolates obtained using this phenotypic marker were exhaustively characterised. The most prevalent clonal complex, among Tet^R^-MRSA non-CC398, was CC1 (46%) and the *spa*-type most frequently found was t127 (38 isolates). Thus, CC1-t127 isolates were identified in 14 out of the 17 participant hospitals. Considering the total of Tet^R^-MRSA isolates (including CC398, characterised in a previous study), CC1 was found in 19% of them [14]. CC1 isolates were identified among Tet^R^ MRSA in other hospitals in the same range as those in our study (12–19% of Tet^R^-MRSA) [7,23].

Some clones related to MRSA CC1, such as the well-known PVL producer USA400, have been broadly described [24]. Among our CC1 isolates, only one CC1-t127 was PVL-positive. Several PVL-negative CC1 clones have also been identified; the Western Australia (WA) MRSA-1 clone was first discovered in Australia in the 1980s, and, since then, these isolates have been detected in several European countries [24]. This PVL-negative clone, like USA400, is not usually associated with multidrug resistance profiles [25]; however, it typically carries *blaZ* and *ermC* antimicrobial resistance genes, and other virulence genes, including *scn* [26]. More recently, another PVL-negative MRSA-CC1 clone (different from the WA-MRSA-1) was identified in humans in Ireland with an alarming MDR profile and mupirocin resistance as a common feature [27]; this clone has been referred to as the European CC1-MRSA-IV clone [28]. These three MRSA CC1 clones share the feature that all of them present with SCC*mec* type IV, despite the fact that other MRSA CC1 clones can present SCC*mec* type V [24], mostly among swine-related isolates [20].

In our study, we found 11 isolates (10 characterised as t127 and one as t1381) that could match the description of the WA-MRSA-1 clone (SCC*mec* IV, PVL-negative, ST1, *blaZ*-positive, *ermC*-positive and *scn*-positive), and one additional t127 isolate with the same features but a PVL producer. These potential WA-MRSA-1 isolates were mupirocin-susceptible and showed resistance to fewer families of antimicrobial agents in relation to the other three isolates (two t127 and one t2207) with features of the MDR-MRSA-CC1 clone (SCC*mec* IV, PVL-negative, and resistance to six–seven different groups of antimicrobial agents, including mupirocin).

Other CCs detected among our Tet^R^-MRSA non-CC398 isolates were as follows: CC5 (11%), CC8 (11%), CC45 (11%), CC88 (3%), CC80 (2%), CC7 (1%) and CC121 (1%). Analysing the use of tetracycline resistance to detect LA-MRSA isolates, in this study we identified clonal lineages previously related to both animals and humans, such as CC1, CC5, CC8 or CC30 [9]. However, other clones that are clearly associated with human infections (as CC45-t1081) [29,30] were also found. In this regard, it was important to analyse the presence of the IEC, since its absence has been associated with an animal origin [7,20]. IEC-negative isolates belonging to CC1, CC5, CC8 and CC30 were identified. These clonal lineages have been found in isolates from very diverse livestock animals (pigs, cattle, horses or poultry) [9,12].

Even though no correlation between pig density and the cases of MRSA CC1 or MRSA t127 or *scn*-negative MRSA in hospitals was found in our study (*p* > 0.05), we cannot discard that this result could be biased by the small number of samples collected. Further studies should be performed, and tetracycline-susceptible isolates should be considered as well.

On the other hand, CC45-t1081 (the second *spa*-type most prevalent in our study) corresponds with the clone known as the Berlin clone [29,30,31,32]. This clone is usually associated with a health care and nosocomial transmission (especially linked to nursing homes) [30,31]. Remarkably, tetracycline resistance was identified in more than 20% of isolates belonging to the Berlin clone in one study carried out in several European countries [33] and in most MRSA CC45 isolates (71%) obtained in one study in Poland [34]. In these studies, this phenotype was remarked for the microorganism’s capacity to acquire multiple antimicrobial resistance determinants. This clone was also worryingly associated with toxigenic isolates [34].

Moreover, two of our t17235 isolates (new *spa*-type detected in this study) corresponded to ST2625. This ST was responsible for an outbreak in a Pediatric Intensive Care Unit in Italy in 2016 [35], and our t17235 isolates presented exactly the same resistance profile as those detected in the Italian hospital: *tetM*, *tetL*, *mecA*, *blaZ* and *aac(6′)-Ie-aph(2″)-Ia* genes. Another clonal lineage detected was CC121 in a PVL-producer isolate with a new *spa*-type (t17234). This CC is becoming an emergent and hypervirulent clone, with more than 90% of CC121 (ST121) isolates carrying the PVL virulence factor [36,37].

A high proportion of MDR phenotypes between the Tet^R^-MRSA non-CC398 isolates studied were detected (86%). Isolates belonging to CC1 did not show a significant increase or decrease in antimicrobial resistance compared to the remaining CCs (*p* > 0.05). Specifically, the rates of resistance genes found in CC1 isolates were: 100% *mecA*, 100% *tetK*, 73% *blaZ*, 56% *ermC*, 29% *msrA*, 11% *ermB*, 9% *ant(4′)-Ia*, 4% *lsaB*, 2% *tetL*, 2% *mupA*, and no isolates with *tetM*, *ermA*, *ermT*, *linA*, *linB*, *vgaA*, *aac(6′)-Ie-aph(2″)-Ia* or *fusB* genes. Interestingly, more than half of the MRSA-CC1-*scn*-negative isolates lacked the *blaZ* gene (52.9%), being a lower rate among MRSA-CC1-*scn*-positive isolates (7.4%) or among the MRSA-*scn*-positive of other non-CC1 lineages (4.8%). These data suggest that the MRSA strains with the *scn* gene (human adaptation marker) carry the *blaZ* gene in most cases, that is frequently absent among the MRSA isolates *scn*-negative (of potential animal origin). In this respect, a high rate of penicillin susceptibility (probably lacking *blaZ*) has been previously detected among animal *S. aureus* isolates [38,39]; moreover, *scn*-negative was a frequent characteristic of clinical methicillin-susceptible *S. aureus blaZ*-negative isolates in a multicentre Spanish study [40]. This potential link of *scn*-negative and *blaZ*-negative characteristics should be evaluated in the future in larger *S. aureus* collections, including MRSA/MSSA as well as *scn*-positive/negative isolates.

The important virulence factor PVL was found in 16% of isolates, and they belonged to very different clonal lineages (CC1, CC8, CC30, CC80, CC88, and CC121). Interestingly, PVL was identified in three IEC-negative isolates. This virulence gene is not frequently found in LA-MRSA isolates, but unfortunately its presence in animal isolates cannot be discounted [9].

Regarding the origin of MRSA-Tet^R^ non-CC398 isolates obtained in this study, it is important to remark that few of them were recovered from invasive infections or from urinary or surgical infections (4.2% each); as a matter of fact, most of them were from SSTI, respiratory tract infections or epidemiological samples (87.5%); this fact suggests that the dissemination routes for these tetracycline-resistant clonal lineages could be respiratory (inhalation) or contact transmission, as has also been indicated for MRSA-CC398 isolates [14]. These routes of transmission facilitate the colonisation of carriers that eventually could develop into cutaneous or respiratory infections.

## 4. Materials and Methods

### 4.1. Selection of Isolates

A collection of 95 Tet^R^-MRSA non-CC398 isolates obtained in a previous study [14] was analysed in the present work. Isolates were recovered from clinical and epidemiological samples during a six-month period (2016) in a multicentre study of 20 participant Spanish hospitals (full names of hospitals in Table 1). In particular, 17 out of the 20 hospitals contributed with Tet^R^-MRSA non-CC398 isolates.

### 4.2. Molecular Typing

The molecular characterisation of *spa*-types (*S. aureus* protein A) was performed by PCR and sequencing for all 95 Tet^R^-MRSA non-CC398 isolates, considering that 38 of them were typed in the previous study as t127-CC1 [18]. The *spa* gene sequences were analysed with Ridom^®^ StaphType software (version 2.2.1). The sequence types (STs) of selected isolates and those with a new *spa*-type were determined by multilocus sequence typing (MLST) as described in https://pubmlst.org/organisms/staphylococcus-aureus (accessed on 1 April 2021). For the remaining isolates, the clonal complex (CC) was assumed according to their *spa*-types. Determination of the SCC*mec* type was performed by PCR for isolates belonging to lineage CC1-t127 [41].

### 4.3. Detection of the Immune Evasion Cluster System (IEC) and the Panton–Valentine Leucocidin (PVL) Genes

The presence of the IEC system was analysed through the detection of the *scn* gene, present in all types of IEC and used as a marker [42]. For the detection of *lukF/lukS* genes, which encode the PVL toxin, all isolates were subjected to PCR technique [43].

### 4.4. Antimicrobial Susceptibility Testing

Susceptibility to 13 antimicrobial agents, in addition to β-lactams and tetracycline, was performed using automatic methods and/or disk diffusion tests for all isolates. The antimicrobials tested were as follows: penicillin, oxacillin/cefoxitin, tetracycline, erythromycin, clindamycin, ciprofloxacin, trimethoprim/sulfamethoxazole, vancomycin, teicoplanin, linezolid, daptomycin, fusidic acid, mupirocin, gentamicin, tobramycin and chloramphenicol. Breakpoints used for interpretation were those according to the Clinical and Laboratory Standards Institute and/or the European Committee on Antimicrobial Susceptibility Testing, depending on hospitals. When a MRSA isolate was resistant to, at least, one agent of three or more different antimicrobial families, a multidrug resistance (MDR) phenotype was associated.

### 4.5. Detection of Antimicrobial Resistance Genes

The presence of the methicillin-resistance gene *mecA* was previously detected and confirmed in all MRSA-Tet^R^ isolates [14]. In isolates with resistance phenotypes, the following antimicrobial resistance genes have been analysed in this study by PCR: *tetM, tetK*, and *tetL* (tetracycline); *ermA, ermB, ermC, ermT, msrA, linA, linB, lsaB* and *vgaA* (macrolides and/or lincosamides), *ant(4′)-Ia* and *aac(6′)-Ie-aph(2″)-Ia* (aminoglycosides), *mupA* (mupirocin) and *fusB* (fusidic acid) [7,41]. Positive and negative controls from the University of La Rioja were used in all PCRs.

### 4.6. Statistical Analysis

Comparison of rates of interest were completed by using Pearson’s Chi-square tests or Fisher’s exact tests when some sample sizes were small (below five isolates). For estimating the association between the Tet^R^-MRSA-CC1, Tet^R^-MRSA-t127 or Tet^R^-MRSA-noCC398-*scn*-negative occurrence in hospitals and the pig density (pigs/km^2^) in adjoining regions, Spearman rank correlations were performed.

All statistical analyses were made with the RStudio program (version 1.1.453) (*p* < 0.05 was considered statistically significant).

## 5. Conclusions

Tetracycline resistance is a good marker for the detection of MRSA CC398 isolates. Nevertheless, other clonal lineages related to livestock animals could also be detected using this marker; in this sense, *scn*-negative MRSA isolates belonging to CC1, CC5, CC8 and CC30 lineages, of potential animal origin, were frequently detected among non-CC398 Tet^R^-MRSA isolates (27.4%). The knowledge of other Tet^R^-MRSA genetic lineages is highly relevant, since most of them were MDR and some of them presented important virulence factors. Moreover, other human CC1 subpopulations (*scn*-positive) as well as the CC45 lineage (associated with facility centres/hospitals) were also detected among Tet^R^-MRSA isolates. The use of tetracycline resistance for detection, not only of CC398, but of other LA-MRSA lineages and other relevant human lineages should be tracked in the future.

## Figures and Tables

**Table 1:** Distribution of non-CC398 Tet^R^-MRSA isolates among hospitals and genetic lineages detected.

Hospital Number ^a^	Number of Isolates					Rates (%)				Density (per km^2^)		*scn*^c^ (Number of Non CC398 Isolates)		Clonal Complex (Number of Non-CC398 Isolates)
	MRSA	Tet^R^-MRSA	Tet^R^-MRSA CC398	Tet^R^-MRSA Non-CC398	Tet^R^-MRSA CC1	Tet^R^-MRSA/ MRSA	Tet^R^-MRSA Non-CC398/Tet^R^-MRSA	Tet^R^-MRSA CC1/Tet^R^-MRSA	Tet^R^-MRSA CC1/Tet^R^-MRSA Non-CC398	Pigs	Inhabitants	+	−	
H1	87	33	27	6	6	38	18	18	100	247.46	717.36	6	0	CC1 (6)
H2	135	20	19	1	0	15	5	0	0	217.68	14.05	1	0	CC45 (1)
H3	328	24	15	9	1	7	38	4	11	217.68	14.05	8	1	CC1 (1), CC45 (8)
H4	251	34	18	16	12	14	47	35	75	142.66	55.20	8	8	CC1 (12), CC5 (1), CC8 (2), CC88 (1)
H5	175	20	9	11	5	11	55	25	45	142.66	55.20	10	1	CC1 (5), CC5 (1), CC8 (1), CC45 (1), ST5427 (1), unknown (2)
H6	76	9	7	2	2	12	22	22	100	142.66	55.20	1	1	CC1 (2)
H7	42	6	3	3	2	14	50	33	67	142.66	55.20	1	2	CC1 (2), CC80 (1)
H8	36	4	4	0	0	11	0	0	0	69.97	9.15	-	-	-
H9	36	2	2	0	0	6	0	0	0	50.89	61.90	-	-	-
H10	206	14	7	7	3	7	50	21	43	50.89	61.90	5	2	CC1 (3), CC8 (1), CC80 (1), CC121 (1), unknown (1)
H11	84	7	3	4	2	8	57	29	50	42.10	138.18	3	1	CC1 (2), CC8 (2)
H12	220	6	3	3	1	3	50	17	33	27.66	25.54	3	0	CC1 (1), CC8 (1), unknown (1)
H13	42	2	0	2	0	5	100	0	0	27.66	25.54	0	2	CC5 (2)
H14	112	6	4	2	1	5	33	17	50	18.31	62.51	0	2	CC1 (1), CC8 (1)
H15	334	5	5	0	0	1	0	0	0	40.99	107.53	-	-	-
H16	130	7	3	4	0	5	57	0	0	30.56	360.18	3	1	CC8 (1), CC88 (1), unknown (2)
H17	315	12	0	12	4	4	100	33	33	20.85	810.66	12	0	CC1 (4), CC5 (2), CC7 (1), ST2625 (2), unknown (3)
H18	277	7	3	4	2	3	57	29	50	20.19	517.95	0	4	CC1 (2), CC5 (1), CC30 (1)
H19	371	13	5	8	2	4	62	15	25	00.45	109.06	7	1	CC1 (2), CC5 (3), CC8 (1), CC30 (1), CC88 (1)
H20	126	1	0	1	1	1	100	100	100	00.45	109.06	1	0	CC1 (1)
Total	3383	232	137 ^b^	95	45	6.8	40.9	13.4	47.4	-	-	69	26	-

^a^ Numeric code used for the analysed hospitals (H). H1: H. Universitari de Vic; H2: H. de Barbastro; H3: H. San Jorge; H4: H. Universitario Miguel Servet; H5: H. Universitario Lozano Blesa; H6: H. Royo Villanova; H7: H. Ernest Lluch Martin; H8: H. de Alcañiz; H9: Clínica Universitaria de Navarra; H10: Complejo Hospitalario de Navarra; H11: H. Virgen Macarena; H12: H. Universitario de Burgos; H13: H. Santiago Apóstol; H14: H. San Pedro; H15: H. Universitario de Álava; H16: H. Universitario de Donostia; H17: H. Universitario Gregorio Marañón; H18: H. de Galdakao; H19: H. Marqués de Valdecilla; H20: H. Sierrallana. ^b^ These strains were previously characterised [14]. ^c^ *scn*: presence or absence of the *scn* gene as marker of the immune evasion cluster (IEC).

**Table 2:** Molecular characterisation of the non-C398 Tet^R^-MRSA collection (n = 95) attending to their *spa*-types and sample origin.

Clonal Complex (CC) (%)	*spa*-Type	No of Isolates	Sample Origin (No of Isolates)	*scn* ^+ g^	PVL^+ h^
CC1 (46%)	t127	38	SSTI ^b^ (17), ES ^c^ (11), RTI ^d^ (5), blood (3), UTI ^e^ (1), SSI ^f^ (1)	23	1
	t174, t693	2	SSTI	2	0
	t1381	2	ES (1), RTI (1)	1	0
	t1784	1	Blood	1	1
	t2207	1	ES	0	0
CC5 (11%)	t002	5	SSTI (2), ES (2), UTI (1)	4	0
	t067	3	SSTI (2), UTI (1)	1	0
	t688	1	RTI	1	0
	t1594	1	ES	1	0
CC7 (1%)	t091	1	SSTI	1	0
CC8 (11%)	t008	1	SSTI	1	1
	t064, t1476	2	RTI	2	0
	t148	6	SSTI (5), ES (1)	3	2
	t2849	1	RTI	0	0
CC30 (2%)	t665	2	SSTI (1), SSI (1)	1	2
CC45 (11%)	t1081	10	SSTI (6), ES (2), SSI (1), RTI (1)	10	0
CC80 (2%)	t044	1	SSTI	1	1
	t088	1	SSTI	1	0
CC88 (3%)	t690	2	RTI	2	2
	t4103	1	UTI	1	1
CC121 (1%)	t17234 ^a^	1	SSTI	1	1
ST2625 (2%)	t17235 ^a^	2	SSTI	2	0
ST5427 ^a^ (1%)	t17236 ^a^	1	SSTI	1	0
Unknown (11%)	t437	1	RTI	1	1
	t992	1	SSI	1	0
	t1354	1	SSTI	1	1
	t1954, t3324	2	SSTI	2	0
	t4725	2	RTI	0	1
	t10419	3	SSTI	3	0

^a^ New *spa*-type or sequence type; ^b^ SSTI: skin and soft tissue infections; ^c^ ES: epidemiological surveillance; ^d^ RTI: respiratory tract infections; ^e^ UTI: urinary tract infections; ^f^ SSI: surgical site infection; ^g^ *scn*^+^: presence of the *scn* gene as marker of the immune evasion cluster (IEC); ^h^ PVL^+^: presence of the genes encoding Panton–Valentine leukocidin.

**Table 3:** Characterisation of tetracycline-resistant CC1 MRSA isolates (n = 44).

*spa*-Type (No of Isolates)	Antimicrobial Resistance (Non Beta-Lactams)		No of *blaZ* Positive Isolates	No of *scn* Negative Isolates	No of PVL Positive Isolates	Origin (No of Isolates) ^c^
	Phenotype ^a^ (No of Isolates)	Genotype ^b^				
t127 (38)	TET (5)	*tetK*	4	1	0	ES (5)
	ERY-TET (6)	*tetL*^1^, *tetK, msrA*	6	1	0	RTI (1), SSTI (5)
	CLI-ERY-TET (17)	*tetK, ermB*^2^, *ermC*^15^, *msrA*^4^, *lsaB*^1^	11	8	1	ES (3), RTI (3), SSTI (7), SSI (1), blood (3)
	CIP-TET (1)	*tetK*	1	0	0	ES (1)
	ERY-CIP-TET (1)	*tetK, msrA*	1	1	0	RTI (1)
	CLI-ERY-CIP-TET (5)	*tetK, ermB*^3^, *ermC, msrA*^1^, *lsaB*^1^	4	2	0	ES (1), SSTI (3), UTI (1)
	TOB-CLI-ERY-TET (1)	*tetK, ermC, ant(4′)-Ia*	1	0	0	SSTI (1)
	TOB-CLI-ERY-MUP-TET (2)	*tetK, ant(4′)-Ia, ermC, mupA* ^1^	1	2	0	ES (1), SSTI (1)
t1381 (2)	TET (1)	*tetK*	0	1	0	RTI (1)
	CLI-ERY-CIP-TET (1)	*tetK, ermC*	1	0	0	RTI (1)
t174 (1)	CIP-TET (1)	*tetK*	1	0	0	SSTI (1)
t693 (1)	TET (1)	*tetK*	0	0	0	SSTI (1)
t1784 (1)	ERY-TOB-TET (1)	*tetK, ant(4′)-Ia, msrA*	1	0	1	Blood (1)
t2207 (1)	CLI-ERY-MUP-CIP-TET (1)	*tetK, ermC*	1	1	0	RTI (1)

^a^ TET, tetracycline; ERY, erythromycin; CLI, clindamycin; CIP, ciprofloxacin; MUP, mupirocin; ^b^ A number in superscript reflects the number of isolates of the group that has the referred characteristic; ^c^ ES: epidemiological surveillance; RTI: respiratory tract infections; UTI: urinary tract infections; SSI: surgical site infection.

**Table 4:** Macrolide-lincosamide resistance phenotypes among TetR-MRSA isolates and resistance genes detected ^a,b^.

Phenotype	*ermA*	*ermB*	*ermC*	*ermT*	*msrA*	*linA*	*linB*	*lsaB*	*vgaA*	No of Isolates
ERY^R^-CLI^S^	−	−	−	−	+	−	−	−	−	19
ERY^R^-cCLI^R^	−	−	+	−	+	−	−	−	−	5
	−	−	+	−	−	−	−	−	−	3
	−	+	+	−	−	−	−	−	−	2
	−	+	−	−	+	−	−	−	−	2
	+	+	−	−	+	+	−	+	−	1
	−	+	−	−	+	−	−	+	−	1
	−	+	+	−	−	−	−	+	−	1
	−	−	+	−	+	+	−	−	−	1
ERY^R^-iCLI^R^	−	−	+	−	−	−	−	−	−	25
	−	−	+	−	+	−	−	−	−	4
	−	−	+	−	+	+	−	−	−	1
	−	+	−	−	+	−	−	−	−	1
	−	+	−	−	+	−	−	+	−	1
	−	−	−	−	−	−	−	−	−	1

^a^ Genes tested: *ermA, ermB, ermC, ermT, msrA, linA, linB, lsaB* and *vgaA*. ^b^ Symbols: +: positive result; −: negative result.

## Data Availability

Not applicable.
